# Purmorphamine Attenuates Neuro-Inflammation and Synaptic Impairments After Hypoxic-Ischemic Injury in Neonatal Mice via Shh Signaling

**DOI:** 10.3389/fphar.2020.00204

**Published:** 2020-03-04

**Authors:** Dexiang Liu, Xuemei Bai, Weiwei Ma, Danqing Xin, Xili Chu, Hongtao Yuan, Jie Qiu, HongFei Ke, Sen Yin, Wenqiang Chen, Zhen Wang

**Affiliations:** ^1^Department of Medical Psychology and Ethics, School of Basic Medicine Sciences, Shandong University, Jinan, China; ^2^Department of Physiology, School of Basic Medical Sciences, Shandong University, Jinan, China; ^3^Qilu Hospital, Shandong University, Jinan, China

**Keywords:** purmorphamine, sonic hedgehog signaling, neuro-inflammation, oxidative stress, synapses, neonatal hypoxia-ischemia

## Abstract

Purmorphamine (PUR), an agonist of the Smoothened (Smo) receptor, has been shown to function as a neuroprotectant in acute experimental ischemic stroke. Its role in hypoxic-ischemic (HI) brain injury in neonatal mice remains unknown. Here we show that PUR attenuated acute brain injury, with a decrease in Bax/Bcl-2 ratio as well as inhibition of caspase-3 activation. These beneficial effects of PUR were associated with suppressing neuro-inflammation and oxidative stress. PUR exerted long-term protective effects upon tissue loss and improved neurobehavioral outcomes as determined at 14 and 28 days post-HI insult. Moreover, PUR increased synaptophysin (Syn) and postsynaptic density (PSD) protein 95 expression in HI-treated mice and attenuated synaptic loss. PUR upregulated the expression of Shh pathway mediators, while suppression of the Shh signaling pathway with cyclopamine (Cyc) reversed these beneficial effects of PUR on HI insult. Our study suggests a therapeutic potential for short-term PUR administration in HI-induced injury as a result of its capacity to exert multiple protective actions upon acute brain injury, long-term memory deficits, and impaired synapses. Moreover, we provide evidence indicating that one of the mechanisms underlying these beneficial effects of PUR involves activation of the Shh signaling pathway.

## Introduction

Perinatal brain injury due to HI is a serious health problem affecting full-term and preterm human neonate and is a contributor to perinatal morbidity and mortality (Volpe, 2012). Effective pharmacological treatments are urgently required to prevent the brain damage and poor long-term outcome.

The Shh signaling pathway serves as a key component in embryonic development and adult stem cell function. After secretion, Shh binds to its receptor Ptch1, which suppresses the Smo receptor. Subsequently, this allows Gli-1/2 to enter the nucleus and initiating the expressions of target genes (Chari and McDonnell, 2007). A number of beneficial effects result from Shh signaling as demonstrated in various pathological states of brain and spinal cord pathology, including acute brain injury (Amankulor et al., 2009), Alzheimer’s disease (Vazin et al., 2014), Parkinson’s disease (Tsuboi and Shults, 2002), stroke (Yu et al., 2017), multiple sclerosis and demyelination (Franco et al., 2008), spinal cord injury (Bambakidis et al., 2010), and HIV-associated neurological disorders (Singh et al., 2016). Complementing these findings are results from reports indicating that activation of the Shh signaling pathway has beneficial effects against brain injury. For example, reductions in spinal cord injury have been reported following the application of recombinant Shh protein or the intravenous hedgehog agonist, Ag11.1 in adult rats (Bambakidis et al., 2003, 2010). In addition, exogenous Shh protein administration exerts angiogenic effects and protects against brain injury from ischemia (Chen et al., 2017).

Purmorphamine is a purine-derivative, small molecule SMO receptor agonist (Sinha and Chen, 2006). In 2002, Schultz et al. demonstrated that PUR induced osteoblast differentiation of multipotent mesenchymal progenitor cells and lineage-committed preosteoblasts (Wu et al., 2002). In addition, PUR enhanced cell survival in HT22 cells in response to an oxidative challenge (Peterson and Turnbull, 2012). PUR has also been shown to promote barrier formation and plays a role in activation of the endogenous anti-inflammatory system within the central nervous system (Alvarez et al., 2011). Recently, PUR was found to protect cortical neurons and restore neurological deficits after ischemic stroke in rats (Chechneva et al., 2014). Work within our laboratory has revealed that PUR exerts neuroprotection against subarachnoid hemorrhage-induced injury in rats (Hu et al., 2016). To the best of our knowledge, the potential effects of PUR within neonatal animals subjected to HI injury remain unknown. In the present study, we examined the influences of a short-term PUR administration on acute brain injury and long-term neurobehavioral dysfunction in neonatal mice subjected to HI injury.

## Materials and Methods

### HI Model and Treatment

The Rice-Vannucci model was used to generate HI injury (Liu et al., 2017). Male C57BL/6J mouse pups on PND 7 were randomly selected. The pups were anesthetized, and the right common carotid artery was isolated and permanently double-ligated. After surgery, the pups were allowed to recover for 1 h and then placed in a hypoxia chamber (humidified 8% O_2_ + 92% N_2_) for 90 min to induce HI injury. Sham controls consisted of anesthetized pups with exposed right carotid arteries.

The PND7 pups were randomized within litters and allocated to one of five groups: (1) Sham + saline (Sham, *n* = 45); (2) HI + saline (HI, *n* = 48); (3) HI + PUR (*n* = 47); (4) HI + PUR + Cyc (Shh antagonist) (*n* = 48); and (5) HI + Cyc (*n* = 48). The initial PUR (10 mg/kg, i.p.) or equal amounts of saline treatment were administered at 24 h after HI injury and followed by daily treatments for the subsequent 3 days. The HI + PUR + Cyc group were pretreated with Cyc (0.5 mg/kg) followed by PUR treatment 30 min later, while the HI + Cyc animals (group 5) were pretreated with Cyc, followed by saline treatment 30 min later. A summary of the experimental schedule is presented in Supplementary Figure S1, while all reagents used in this study are contained within Table 1.

**TABLE 1:** All reagents used in this study.

Primary antibodies	Dilution	Cat. No.	Producer
**1.1 Antibodies used for Western blot analysis**			
Anti-β-actin	1:1000	TA-09	Zhongshan Golden Bridge Biotechnology, Beijing, China
Anti-Bax	1:1000	50599-2-Ig	Proteintech Group, United States
Anti-Bcl-2	1:1000	12789-1-AP	Proteintech Group, United States
Anti-Cleaved caspase-3	1:500	Asp175, #9661	Cell Signaling, Beverly, MA, United States
Anti-Caspase-3	1:1000	19677-1-AP	Proteintech Group, United States
Anti-Nrf2	1:500	16396-1-AP	Proteintech Group, United States
Anti-Syn	1:1000	D35E4, #5461	Cell Signaling, Beverly, MA, United States
Anti-PSD95	1:1000	D74D3, #3409	Cell Signaling, Beverly, MA, United States
Anti-Shh	1:1000	20697-1-AP	Proteintech Group, United States
Anti-Ptch	1:500	ab53715	Abcam, Cambridge, United Kingdom
Anti-Gli-1	1:500	ab151796	Abcam, Cambridge, United Kingdom
**1.2 Antibodies used for immunofluorescence and immunohistochemistry staining**			
Anti-NeuN	1:100	ab104224	Abcam, Cambridge, United Kingdom
Anti-Syn (mouse)	1:100	60191-1-Ig	Proteintech Group, United States
Anti-PSD95 (rabbit)	1:100	20665-1-AP	Proteintech Group, United States
Anti-Cleaved caspase-3	1:100	Asp175, #9661	Cell Signaling, Beverly, MA, United States
Anti-Iba-1	1:100	GTX632426	GeneTex, Inc.,Irvine, CA, United States
**1.3 Other major reagents used in this study**			
Purmorphamine			Selleck Chemicals (Houston, TX, United States)
Cyclopamine			Selleck Chemicals (Houston, TX, United States)
Radioimmunoprecipitation assay (RIPA)			Beyotime Institute of Biotechnology, Jiangsu, China
Phenylmethane-sulfonyl fluoride (PMSF)			Beyotime Institute of Biotechnology, Jiangsu, China
PhosSTOP phosphatase inhibitor			Roche Diagnostics Gmbh, Indianapolis, IN, United States
BCA protein assay kit			CWBIO, Haimen, Jiangsu, China
5 × loading buffer			Beyotime Institute of Biotechnology, Jiangsu, China
PVDF membranes			Millipore, United States
Enhanced chemiluminescence			Millipore, United States
TRIzol reagent			CWBIO, Haimen, Jiangsu, China
RevertAid First Strand cDNA Synthesis Kit			Thermo scientific, Waltham, MA, United States
GolView			Beijing Solarbio Science and Technology Co, China
4,6-Diamidino-2-phenylindole (DAPI)			Beyotime Institute of Biotechnology, Jiangsu, China
Dihydroethiduim			Beyotime Institute of Biotechnology, Jiangsu, China
Terminal deoxynucleotidyl transferase dUTP-mediated nick end labeling (TUNEL) Apoptosis Detection kit			KeyGen Bio Tech Co. (Nanjing, China).
Rhodamine (TRITC)-conjugated goat anti-rabbit IgG			Proteintech Group, United States
Fluorescein (FITC)-conjugated affinipure goat anti-mouse IgG			Proteintech Group, United States
Peroxidase-conjugated goat anti-rabbit/mouse IgG			Zhongshan Golden Bridge Biotechnology
2,3,5-Triphenyltetrazolium chloride monohydrate (TTC)			Sigma–Aldrich, St. Louis, MO, United States
Cresyl violet acetate			Sigma–Aldrich, St. Louis, MO, United States
APC anti-mouse CD45			Biolegend (103112)
FITC anti-mouse/human CD11b			Biolegend (101206)

The animal experiments were performed based on the International Guiding Principles for Animal Research provided by the Council for International Organizations of Medical Sciences (CIOMS), and procedures were approved by the Animal Ethical and Welfare Committee of Shandong University. Participants who worked with the animal models were trained according to Institutional Animal Care and Use Committee Guidebook (IACUC) rules.

### Behavioral Testing

Hypoxic-ischemic insult led to hyperactivity at PND21, but no differences was found in spontaneous or in open-field activity at PND 90 (Lubics et al., 2005). Rats at PND35 appeared adult-like learning during the training trials in MWM (Wood et al., 2003). In this study, the anxiety-like behaviors and the cognitive functions were evaluated at PND23 (14-days post-HI) and PND35 (28-days post-HI), respectively.

### Open Field Test

The exploratory and anxiety-like behaviors of animals were assessed at 14 days post-HI in an open field test as described previously (Liu et al., 2017). Each mouse was placed in the corner of the box and allowed 5 min of exploration. The activities of crossings, rearings, and grooming, and the time in center were recorded for each minute of the test.

### Morris Water Maze (MWM)

The spatial reference memory at 28 days post-HI was assessed using the MWM test as previously described (Liu et al., 2017). On training trials, the time required to locate and climb onto the platform (escape latency) was measured for 5 consecutive days (four trials/day). On probe trial (day 6), the time to reach the original platform in the target zone and total time traveled in the target zone were recorded.

### Evaluation of Neuropathological Injury

For histological endpoint determinations, brains were harvested, fixed in 4% formaldehyde, and then sliced into 5 μm coronal sections in the region containing the infarct lesion (between −1.60 and −2.00 mm from bregma). Slices were then analyzed using Nissl staining, TUNEL staining, and immunofluorescence or immunohistochemical imaging (Liu et al., 2017). Analyses of the images were performed using the Image-Pro Plus 6.0 software by an investigator blinded as to experimental group assignments. The number of active caspase-3/NeuN double positive cells, Nrf-2 positive cells, and TUNEL-positive cells were counted within randomly selected peri-infarct areas (within 300 μm to the infarct). The number of Iba-1 positive cells was measured in the core region of the infarct at × 20 magnifications. Activated microglia scores were assigned as previously described (Table 2) (Thei et al., 2018).

**TABLE 2:** Semi-quantitative scores for Iba-1 staining.

Score	Microglial appearance
0	No activation
1	Foci of non-ramified active microglia
2	<50% coverage of active microglia
3	Widespread active and predominantly phagocytic microglia
4	Total phagocytic activation

### Nissl Staining

For Nissl quantification of brain atrophy, brain sections from each group described above (*N* = 4 mice/group) were incubated with 0.5% cresyl violet acetate for 20 min to compare hemispheric atrophy among the groups. The hemispheric lesion volume was calculated using the following formula:

Infarct⁢ration%=(contralateral⁢area-ispiliateral⁢area)/contralateral⁢area×100%

### Infarct Ratio Measurement

After 3 days post-HI, mice were sacrificed and transcardially perfused with PBS to remove intravascular blood. The brain was harvested and divided them into four coronal sections. Each section was incubated within 2% of 2, 3, 5-TTC monohydrate at 37°C for 20 min. The software, Image-Pro Plus 6.0 was used for the summation of the volume of each section. The infarct ratio was calculated using the following formula:

$$\mathrm{Infarct}\,\mathrm{volume}\%=\frac{\begin{array}{c} \mathrm{contralateral}\,\mathrm{hemispheric}\,\mathrm{volume}\\ -\;\mathrm{ipsilateral}\,\mathrm{non}\,\text{-}\,\mathrm{infarcted}\,\mathrm{volume} \end{array}}{\mathrm{contralateral}\,\mathrm{hemispheric}\,\mathrm{volume}}\times 100\%$$

### Transmission Electron Microscopy (TEM)

The preparation method for transmission electron microscopy (TEM) analysis was similar to that described previously (Liu et al., 2017). The cortices from the ipsilateral hemisphere were fixed in 2.5% glutaraldehyde for 2 h, washed with PBS, fixed in 1% osmium tetroxide for 2 h, dehydrated in a graded series of ethanol, embedded in epoxy resin. Ultrathin (50 nm thickness) sections were obtained by Ultra microtome (EM UC 7, Leica, Germany), stained with uranyl acetate. The sections were photographed using Hitachi H-7500 TEM. In the present study, asymmetric synapses were examined for synaptic measurement (Xiao et al., 2014). Asymmetric synapses exhibit the following characteristics: (1) typical asymmetric interface, i.e., the thickness of postsynaptic membrane is much bigger than that of presynaptic membrane; (2) PSD in the postsynaptic membrane; (3) round and clear synaptic vesicles. Six images per animal (20,000×) were used to observe the number of synapses (*n* = 4 each group). The number of synapses was expressed as the average of all the synapses in each photo taken at 20,000×. The synaptic cleft and the thickness of PSD was described in each photo taken at 20,000×.

### Western Blot Analysis

Animals were decapitated after anesthesia at 3, 14, and 28 days after HI insult. Isolated cortices from the right hemisphere were separated and frozen at −80°C. Frozen tissue was cut into small pieces and homogenized in ice-cold RIPA lysis buffer containing 1% PMSF and 0.05% (vol/vol) PhosSTOP phosphatase inhibitor. After centrifugation at 10,000 × *g* for at 4°C 10 min, the supernatant was collected, and then measured the protein concentration using a BCA protein assay kit. The tissue samples were mixed with 5 × loading buffer and boiled for 10 min at 100°C. The proteins were loaded onto a 4–20% gradient polyacrylamide gel, electrophoretically transferred to polyvinylidene difluoride membrane, and probed with the following primary antibodies (all antibody information is presented in Table 1).

### DHE Analysis

For determinations of ROS production in brain sections, brains were harvested, fixed in 4% formaldehyde, and then sliced into 12 μm coronal sections in the region containing the infarct lesion (between −1.60 and −2.00 mm from bregma). The frozen coronal sections from the ipsilateral hemisphere were stained with dihydroethiduim (DHE) as previously described (Wang et al., 2017). Briefly, coronal sections were stained with 10 μM DHE for 30 min. After rinsing and mounting, the fluorescence images were captured with use of fluorescent microscopy (BX51; Olympus, Tokyo, Japan). The DHE-staining results were pixilated and quantified using the Image-pro plus image analysis system.

### Flow Cytometric Analysis

Single cells were prepared as previously described (Henry et al., 2009; Xin et al., 2018) with minor modifications. Briefly, the ipsilateral cortex was quickly removed and immersed into PBS containing 0.2% BSA (w/v) on ice. The cortices of each group were gently ground on the 70 μm cell strainer at 4°C. Then, homogenates were filtered by passing through a 70 μm cell strainer. The cell suspension was centrifuged at 400 × g for 10 min to obtain precipitation, and the cells were re-suspended with 40% Percoll solution, followed by the addition of 75% Percoll solution at the bottom, and centrifuged at 500 × g for 20 min. Cells were collected from two density gradient interfaces and incubated by these antibodies for 30 min at 4°C: anti-mouse CD45-APC (10311, Biolegend) or anti-mouse CD11b-FITC (101206, Biolegend), to evaluate the populations of infiltrating monocytes/neutrophils (CD11b^+^/CD45^high^ cells). Flow cytometric analysis was performed with use of FACS flow cytometer C6 (BD Biosciences). CD11b^+^ cell populations were further gated for CD45 expression and divided into a low (microglia) and high population (infiltrating monocytes/neutrophils).

### Statistical Analysis

Data were analyzed by the SPSS software program. All values presented are expressed as the mean ± standard deviation. Data from the training trials in the MWM were averaged for each mouse (total data/total number of trials per day). Daily performance scores in the MWM tests were evaluated with use of a repeated-measures two-way analysis of variance (ANOVA) with “days” as the within-subject factor and “groups” as the between-subject factor. Daily scores of different groups in MWM tests were compared using one-way ANOVA, following by *post hoc* Bonferroni corrections for multiple comparisons. Correlation analysis between Bax/Bcl-2 ratio and TUNEL counts, between Syn/PSD protein 95 staining and escape latencies from day 1 to day 6 in MWM test was performed with Pearson correlation test. Unless otherwise indicated, other data were analyzed using the one-way ANOVA, and *post hoc* analysis between groups was performed using the Bonferroni corrections for multiple comparisons in this study. A *p*-value < 0.05 was required for results to be considered statistically significant.

## Results

### PUR Prevents HI-Induced Brain Injury

In the preliminary pilot study, we tested the effects of PUR at 1, 5, and 10 mg/kg upon infarct volumes and edema. PUR treatment at 5 and 10 mg/kg reduced infarct volumes (*p* < 0.05, *p* < 0.001, respectively), PUR treatment at 10 mg/kg reduced edema (*p* < 0.001), compared with those in the HI group at 72 h post-HI. From these studies, we established that the 10 mg/kg dose exerted remarkable beneficial effects in the absence of any toxicity (data not shown) and therefore we chose this dose for the subsequent experimental studies.

**FIGURE 1:**
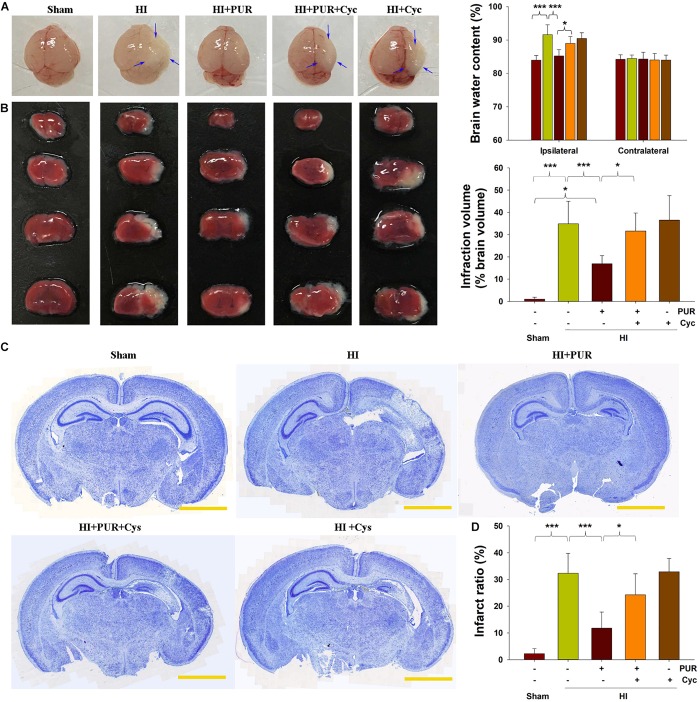
Effects of PUR on HI-induced brain injury at 72 h post-HI insult. **(A)** Representative images of brain sections from each group. Arrows indicate edematous change in morphology within the ipsilateral hemisphere. Quantification of brain water content within ipsilateral and contralateral brain hemispheres was measured (*N* = 6/group). **(B)** Representative TTC stained brain sections of each group. Coronal sections were processed with 2% TTC staining for 20 min, and infract volume quantification was determined: infarct volume = (contralateral hemispheric volume – ipsilateral non-infarcted volume)/contralateral hemispheric volume. Quantitative analysis of lesion volume of each group (*N* = 6/group). **(C)** Representative Nissl staining of coronal sections of each group. Brain sections from each group (four sections/mouse) were incubated with 0.5% cresyl violet acetate for 20 min. Nissl staining measurements were analyzed with use of Image-Pro Plus 6.0 software. Scale bar = 2 cm. **(D)** Quantification of amount of tissue loss of each group. Values represent the mean ± SD with *N* = 6/group. Values represent the mean ± SD, *p < 0.05, ***p < 0.001 according to ANOVA with Bonferroni correction.

As shown in Figure 1A, HI insult led to a remarkable edematous condition and significantly increased the water content within the ipsilateral side at 72 h following HI. PUR treatment markedly reduced this HI-induced brain edema (*p* < 0.001) as compared with vehicle-HI group.

Results from TTC and Nissl stainings showed that the significant increases in brain infarction areas (*p* < 0.001, Figure 1B) and tissue loss (*p* < 0.001, Figures 1C,D) were present within the ipsilateral side at 72 h post-HI. Treatment with PUR, blocked HI-induced brain infarction (*p* < 0.001, Figure 1B) and tissue loss (*p* < 0.001, Figures 1C,D).

Next, we used TEM to confirm neural damage. The neurons in Sham group appeared normal, while neurons in the ipsilateral side of HI group exhibited severely ultrastructural damage at 72 h post-insult. The cytoplasm vacuolization, irregular nuclei, and discontinuous cell membrane were found in some areas. Some vacuoles were shown in mitochondria, and ER exhibited remarkable swelling. However, post-treatment with PUR following HI insult relieved this morphological damage. The ultra-microstructure of neuron in HI + PUR group was similar to those of Sham group (Supplementary Figure S2).

These beneficial effects of PUR on edema, infarct volume, and morphological damage were significantly blocked when mice were pretreated with Cyc combined with PUR. Brain injury in animals pretreated with Cyc alone was not more severe than that in the HI group (Figure 1).

### PUR Decreases TUNEL-Positive Cells and Apoptotic Markers

**FIGURE 2:**
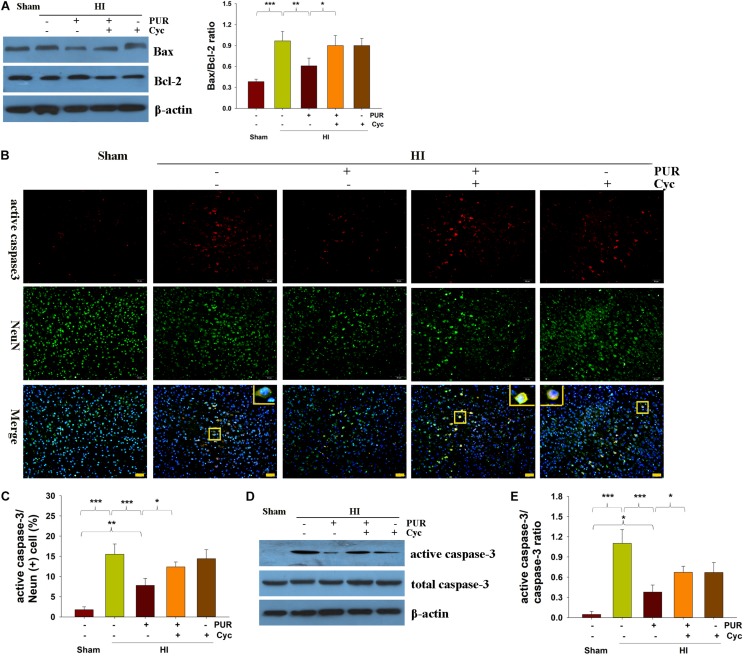
PUR alleviates HI-induced apoptosis at 72 h post-HI insult. **(A)** Levels of Bax and Bcl-2 within the ipsilateral cortex were measured with use of western blotting. Quantification of protein levels of Bax and Bcl-2 were determined with use of Image-Pro Plus 6⋅0 (*N* = 4/group). **(B)** Double immunofluorescent staining of active caspase-3 (red) and NeuN (green) within the ipsilateral cortex. Scale bar = 50 μm. **(C)** Quantification of active caspase-3/NeuN-positive cells (*N* = 4/group). Six images (20×) were captured randomly for each section per animal. **(D)** Immunoblotting analysis of active caspase-3, caspase-3 within the ipsilateral cortex. **(E)** Image-Pro Plus 6.0 was used to quantify protein levels of caspase-3 and active caspase-3. Results are expressed as the ratio of active caspase-3/caspase-3 (*N* = 4/group). Values represent the mean ± SD, *p < 0.05, **p < 0.01, ***p < 0.001 according to ANOVA with Bonferroni correction.

Results from TUNEL staining revealed that PUR treatment significantly reduced cell apoptosis following HI injury (*p* < 0.01, Supplementary Figure S3). HI insult remarkably increased the Bax/Bcl-2 ratio at protein level, while PUR treatment prevented these effects (Figure 2A). Cyc administration reversed the effect of PUR on apoptosis and Bax/Bcl-2 ratio after HI injury (Figure 2). Pearson correlational analysis revealed that Bax/Bcl-2 ratio in protein were positively associated with TUNEL counts following HI insult in mice (*r* = 0.895^∗∗^, *p* < 0.001).

Active caspase-3/NeuN double staining showed that the number of apoptotic neurons in the HI group was markedly increased as compared with that in the Sham group (*p* < 0.001), while PUR treatment significantly decreased the number of active caspase-3/NeuN positive-neurons as compared to vehicle-treated HI mice (*p* < 0.01, Figures 2B,C). Following HI insult, levels of active caspase-3 in the ipsilateral cortex were increased compared with that of the Sham group (*p* < 0.001), while active caspase-3 was decreased in the PUR treatment group (*p* < 0.001) as determined using Western blotting analysis (Figure 2D). These beneficial effects of PUR in decreasing HI-induced caspase-3 activation in mice were blocked by Cyc administration (Figure 2), suggesting that PUR suppressed HI-induced caspase-dependent apoptosis.

### The Role of PUR on the Shh Signaling Pathway in HI Injury

**FIGURE 3:**
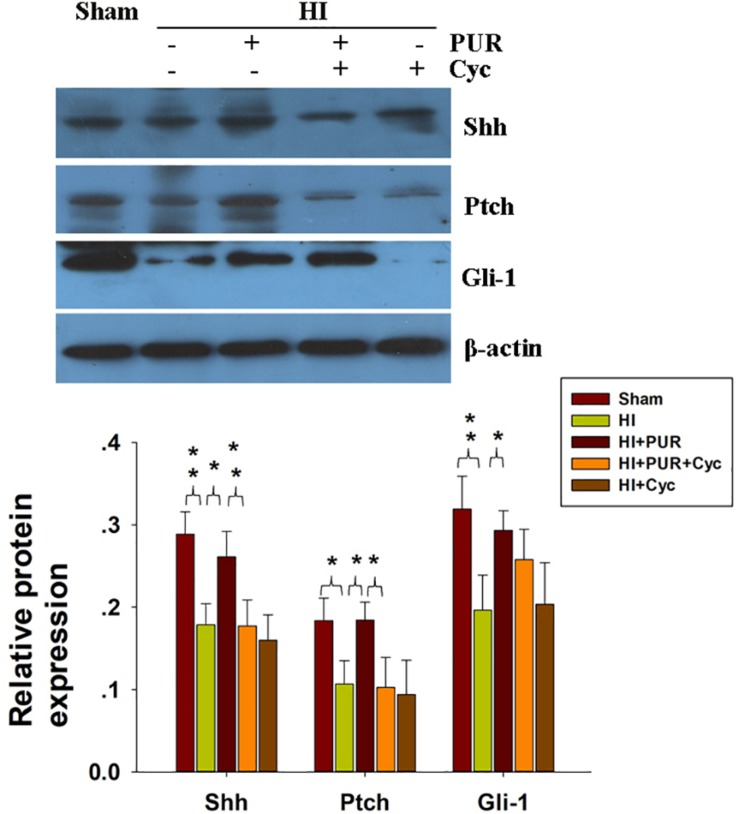
Effects of PUR administration on the Shh pathway. Quantification of Shh, Gli-1, and Ptch was determined with use of western blot as performed at 72 h post-HI. Quantification of protein levels of Shh, Gli-1, and Ptch was determined with use of Image-Pro Plus 6.0 (*N* = 4/group). Values represent the mean ± SD, *p < 0.05, **p < 0.01 according to ANOVA with Bonferroni correction.

Hypoxic-ischemic exposure produced a decrease in the protein expressions of Shh (*p* < 0.01), Ptch (*p* < 0.05), and Gli-1 (*p* < 0.01). PUR treatment reversed HI-downregualted Shh (*p* < 0.05), Ptch (*p* < 0.05), and Gli-1 (*p* < 0.05) expressions (Figure 3). These effects of PUR on the Shh pathway following HI exposure were partially blocked by Cyc pre-treatment (Figure 3).

### PUR Suppressed Neuro-Inflammation Following HI Insult

**FIGURE 4:**
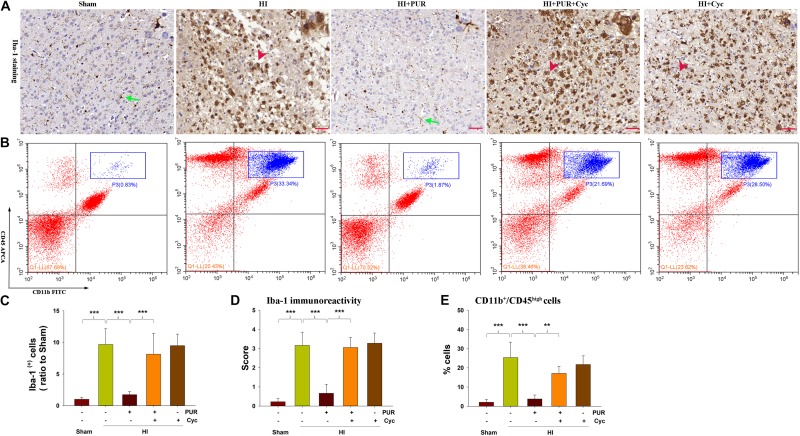
PUR suppressed microglial activation and inflammatory gene expression. **(A)** Expression of Iba-1 within the ipsilateral cortex was examined at 3 days after HI with use of immunohistochemical staining. Green arrow—resting microglia (ramified microglia) and red arrowhead—activated microglia (amoeboid shape). **(B)** Representative flow cytometric lots of CD11b^+^/CD45^high^ cells within ipsilateral cortex. The infliting monocytes/neutrophils in the upper right quadrant (blue rectangle) (CD11b^+^/CD45^high^). **(C)** Quantification of Iba-1^+^ cell numbers in infarct core regions. *N* = 4/group. **(D)** Quantitative assessment of PUR effect on microglial activation score. *N* = 4/group. **(E)** Quantification of CD11b^+^/CD45^high^ cell numbers in ipsilateral cortex. *N* = 4/group. Values represent the mean ± SD, **p < 0.01, ***p < 0.001 according to ANOVA with Bonferroni correction.

Increased Iba-1 labeling cells were observed in the core region of the infarct (*p* < 0.001) following HI, with most of the microglia exhibiting a rounded amoeboid-like appearance. However, PUR treatment decreased HI-increased Iba-1 labeling cells (*p* < 0.001) (Figures 4A,C), with most of the microglia exhibiting a resting phenotype based on microglial activation score (*p* < 0.001) (Figure 4D).

In the HI brain, there is also a considerable contribution of infiltrating peripheral immune cells to the brain after injury (Hellstrom Erkenstam et al., 2016). Next, we investigated the effect of PUR administration on infiltrating monocytes/neutrophils (CD11b^+^/CD45^high^ cells). The result showed that HI exposure remarkedly increased the number of infiltrating monocytes/neutrophils (CD11b^+^/CD45^high^ cells) (*p* < 0.001) within the ipsilateral cortex at 72 h following injury, while PUR administration significantly attenuated these increases (*p* < 0.001) (Figures 4B,E).

### Effects of PUR on Oxidative Defenses After HI Insult

**FIGURE 5:**
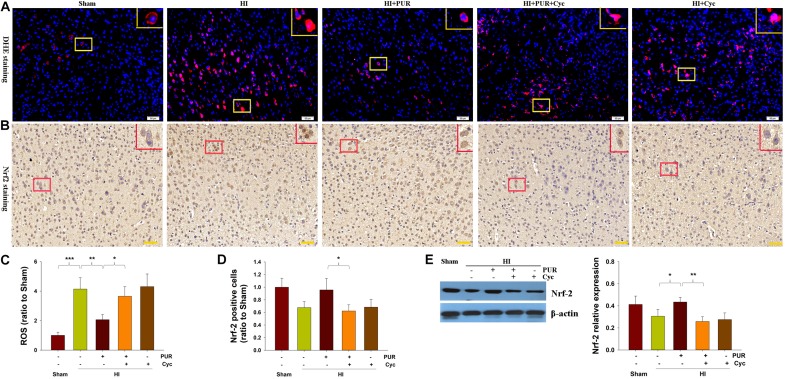
Effect of PUR on ROS level and Nrf2 expression at 72 h post-HI insult. **(A)** The ipsilateral cortex was processed for ROS analysis. Frozen cortex sections were stained for 30 min with DHE to obtain quantitative data of relative fluorescence. Scale bar = 50 μm. **(B)** Immunohistochemical staining of Nrf2 expression within the ipsilateral cortex. Scale bar = 50 μm. **(C)** Quantification of ROS was determined with use of Image-Pro Plus image analysis system. Values are expressed relative to those of the Sham group (*N* = 4/group). **(D)** Quantitative analysis of the number of Nrf2-positive cells (stained in brown). Values are expressed relative to those of the Sham group (*N* = 4/group). Six images (20×) were captured randomly for each section per animal. **(E)** Western blotting was used to measure levels of Nrf-2 within the ipsilateral cortex. Quantification of protein levels of Nrf-2, as determined with use of Image-Pro Plus 6⋅0 (*N* = 4/group). Values represent the mean ± SD, *p < 0.05, **p < 0.01, ***p < 0.001 according to ANOVA with Bonferroni correction.

The ROS content was substantially increased in the ipsilateral cortex at 72 h post-HI injury with DHE staining, whereas PUR treatment suppressed this HI-induced ROS production (*p* < 0.01, Figures 5A,C). Western blot analysis showed that Nrf-2 protein in the ipsilateral cortex was slightly downregulated following HI injury. PUR treatment resulted in significantly higher levels of Nrf-2 expression than that observed within the vehicle-HI group (*p* < 0.05, Figure 5E). These results were corroborated by the data obtained from immunohistochemical analysis (Figures 5B,D). Pre-treatment with Cyc decreased Nrf-2 levels as compared with those observed in the HI + PUR group (Figures 5B,E).

### Effects of PUR on Synaptic Remodeling at 3, 14, and 28 Days After HI

**FIGURE 6:**
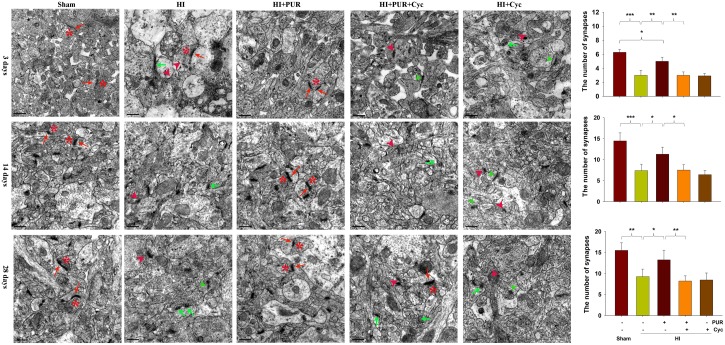
Effects of PUR on synaptic damage post-HI insult. Representative electron micrographs images indicating *: presynaptic vesicles, red arrow: postsynaptic partners, green arrow: few vesicles but no postsynaptic partner, red arrowhead: destroyed synapses, green arrowhead: widened synaptic clefts. Quantification of the number of synapses at 3, 14, and 28 days post-HI insult (*N* = 4/group). The number of synapses was expressed as the average of all synapses present in each photo taken at 20,000×.

As observed using TEM, synaptic density was increased at PND21 and PND35 compared with that at PND10 in each group (Figure 6). HI exposure reduced the numeric synaptic densities at 3, 14, and 28 days post-HI insult (*p* < 0.001, *p* < 0.001, *p* < 0.01, respectively; Figure 6). Synaptic clefts were widened in response to HI. A few synapses were completely destroyed by HI insult. However, in response to PUR treatment, no significant differences were identified in synaptic ultra-structures in the HI as compared with that of the Sham group (Figure 6).

**FIGURE 7:**
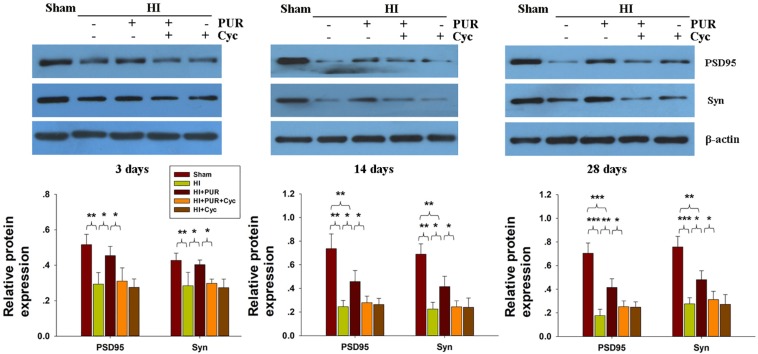
Effects of PUR on synaptophysin (Syn) and PSD95 expression post-HI insult. Immunoblotting analysis of Syn and PSD95 within the ipsilateral cortex. Quantification of expression levels of Syn and PSD95 as determined with use of Image-Pro Plus 6.0 (*N* = 4/group). Values represent the mean ± SD, *p < 0.05, **p < 0.01, ***p < 0.001 according to ANOVA with Bonferroni correction.

Within the ipsilateral cortex, expression levels of Syn and PSD95 proteins were substantially downregulated at 3, 14, and 28 days after HI insult (Supplementary Figure S4). PUR treatment significantly increased the expression of Syn and PSD95 at 3, 14, and 28 days after HI insult, compared with HI group (Figure 7). Cyc combined with PUR markedly inhibited these beneficial effects of PUR on Syn and PSD95 expression (Figure 7).

### PUR Ameliorated Brain Atrophy and Locomotor Activity 2 Weeks After HI

**FIGURE 8:**
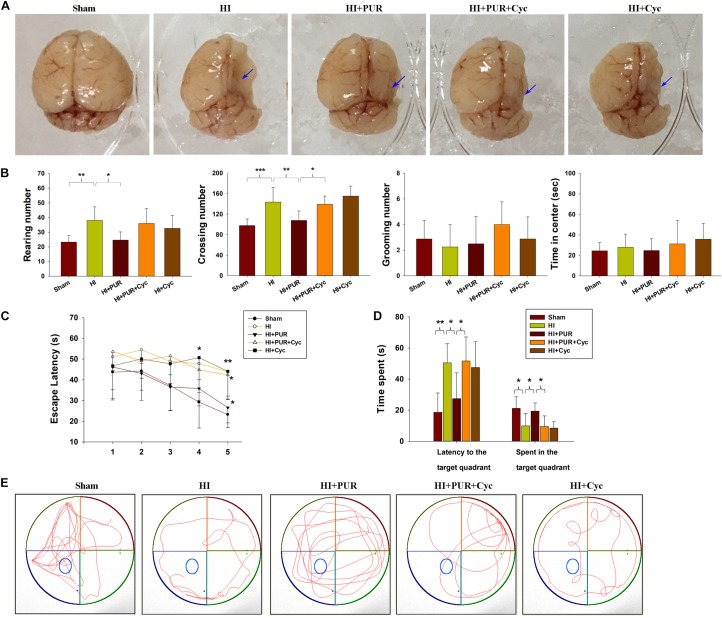
Effects of PUR on brain atrophy and behavioral changes following HI insult. **(A)** Representative morphology of the brain of each group at 14 days post-HI. Arrows indicate atrophy within the ipsilateral hemisphere. **(B)** Locomotion was measured within an open field test (*N* = 8/group). Each mouse was placed in the corner of the box and allowed 5 min of exploration. The following behaviors were recorded for each mouse: number of squares crossed (crossing), frequency of standing on hind limbs (rearing), number of times the animal was observed grooming their face, licking/cleaning and scratching various parts of the body (grooming), and amount of time traveled in the center area of the maze. **(C)** During each acquisition trial in the MWM test, all the animals were allowed 60 s per trial to locate the escape platform, which was submerged 2 cm below the water surface and located in the center of quadrant III. The time required to locate and climb onto the platform (escape latency) was measured for 5 consecutive days (four trials/day). **(D)** On probe trial (day 6), the platform was removed from the tank. Animals were placed in quadrant IV and their swimming path was traced for the following 60 s of the test. The time to reach the original platform in the target zone and total time traveled in the target zone were recorded. **(E)** Display of tracks of all groups on day 6 (*N* = 8/group). Values represent the mean ± SD, *p < 0.05, **p < 0.01, ***p < 0.001 daily scores were compared using ANOVA with Bonferroni correction.

Substantial brain atrophy and asymmetry were found 14 days after HI exposure. Treatment with PUR partially alleviated this damage (Figure 8A). At the same time, HI insult resulted in a large area of tissue loss within the ipsilateral cortex, PUR treatment was able to prevent HI-induced tissue loss (*p* < 0.01, Supplementary Figure S5) as indicated by Nissl staining.

With regard to anxiety-like behaviors, an overall statistically significant difference among the groups was obtained for the number of crossings (*p* < 0.001) and rearings (*p* < 0.01), but grooming (*p* > 0.05) and time in the center (*p* > 0.05) failed to show any overall significant differences among the groups. *Post hoc* analysis revealed that exposure to HI substantially increased crossing (*p* < 0.001) and rearing (*p* < 0.01) scores, while treatment with PUR showed significantly lowered crossing (*p* < 0.01) and rearing (*p* < 0.05) scores as compared with that of the HI group. These beneficial effects of PUR on brain damage and anxiety-like behaviors at PND 21 after HI were partially reversed by Cyc pre-treatment (Figure 8B).

### Effects of PUR on MWM Test Performance After HI

Many studies demonstrated that HI injured animals showed deficits on a long-term reference memory in MWM test (Balduini et al., 2000). In the study, mean latencies declined progressively from training days 1 to day 5 in each group [*F*(4,35) = 11.634, *p* < 0.001] and no significant interaction between training days and groups was present [*F*(4,35 = 1.236, *p* > 0.05] by repeated measures ANOVA. These data revealed that all mice showed the same improvements in spatial learning and memory over time, regardless of previous treatment. In the initial 3 days of testing, no statistically significant differences in latencies were obtained among the groups (*p* > 0.05). However, on day 4 and 5, HI animals spent significantly longer time to locate the platform as compared with the Sham group (*p* < 0.05, *p* < 0.01, respectively, Figure 8C). In response to PUR treatment, a progressive reduction in escape latencies was observed with differences being statistically significant on day 5 (*p* < 0.05, Figure 8C).

In the probe trial, all mice were swimming mostly in quadrant III, where the hidden platform was originally located. The HI group spent significantly less time in quadrant III when compared to the Sham group (*p* < 0.05, Figures 8D,E). However, mice treated with PUR spent significantly increased amounts of time in the target zone as compared with that of the HI group (*p* < 0.05, Figures 8D,E). Significantly decreased escape latencies were observed in the PUR in comparison to that of the HI group (*p* < 0.05, Figures 8D,E). Pre-treatment with Cyc significantly blocked these effects of PUR upon MWM test results. Pearson correlational analysis showed that Syn/PSD95 staining was not associated with escape latencies from day 1 to day 6 in MWM test (Table 3).

**TABLE 3:** Pearson correlational analysis between Syn/PSD95 labeling with escape latencies from day1 to day 6 in MWM test.

	Syn/PSD95 labeling	Escape latencies in day 1	Escape latencies in day 2	Escape latencies in day 3	Escape latencies in day 4	Escape latencies in day 5	Escape latencies in day 6
Syn/PSD95 labeling	*r*^2^ = 1	*r*^2^ = −0.213	*r*^2^ = −0.215	*r*^2^ = 0.209	*r*^2^ = 0.035	*r*^2^ = −0.242	*r*^2^ = −0.442
		*p* = 0.368	*p* = 0.363	*p* = 0.376	*p* = 0.882	*p* = 0.304	*p* = 0.051

## Discussion

In the present study, we demonstrate that treatment with PUR following HI insult protected against acute brain injury and long-term memory and learning deficits in neonatal mice. Some of these beneficial effects of PUR could be attributed to the attenuation of HI-induced synaptic impairments, the reduction in neuro-inflammation and ROS levels, as well as the involvement of the Shh signaling pathway.

### Neuronal Cell Death and Neuro-Inflammation Were Attenuated Following PUR Treatment

The Shh pathway plays a key role in tissue repair after ischemic damage, which has been demonstrated within the heart (Kusano et al., 2004), brain (Chen et al., 2017), and skeletal muscle (Pola et al., 2003). Shh signaling triggered by resveratrol reduced infarct volume and reversed neurological deficits after stroke (Yu et al., 2017). Additionally, Shh pathway activation by Shh agonist improved behavioral deficits resulting from focal cortical ischemic injury (Jin et al., 2015). Recently, PUR was found to participate in neuroprotective effects against ischemic injury, which were reported to be independent of the Shh signaling pathway (Chechneva et al., 2014). Previous work within our laboratory has indicated that PUR provided neuroprotection against subarachnoid hemorrhage-induced injury (Hu et al., 2016). However, only limited information currently exists in support of PUR neuroprotection and its mechanisms, which may involve anti-inflammatory, anti-apoptotic, and/or pro-angiogenic processes after ischemic insult (Chechneva et al., 2014; Chechneva and Deng, 2015). In addition, immediate microglial activation occurs following HI and mediates brain injury (Hagberg et al., 2015; Shao et al., 2017). Activated microglia mainly contributes to neuro-inflammation and releases inflammatory mediators including tumor necrosis factor-α and interleukin-1β, leading to neuronal dead and excitotoxic injury (Mallard et al., 2018). Pharmacologic inhibition of microglial activation suppresses HI-induced neuronal apoptosis (Arvin et al., 2002; Li et al., 2014). In this study, PUR treatment markedly suppressed microglia activation and inflammatory cytokines levels, and reduced infarct size and neuronal apoptosis after HI. Taken together, blocking microglial activation may contribute to the neuroprotective effects of PUR in immature brain in mice following HI.

The capacity for PUR to exert antioxidant effects in HI, as revealed by significantly decreasing HI-induced ROS levels, was consistent with a previous study (Hu et al., 2016). A number of antioxidants, including allopurinol, erythropoietin, resveratrol, and omega-3 fatty acids, have been shown to exhibit neuroprotective properties in different animal models of perinatal HI brain injury (Peeters-Scholte et al., 2003; Arteaga et al., 2017; Revuelta et al., 2017). In this study, PUR minimized oxidative stress associated with HI insult, as indicated with increased Nrf-2 expression levels. Nrf-2 is an redox-sensitive transcription factor that mainly mediates the expression of antioxidant enzymes, including heme oxygenase-1 and NAD(P)H:quinone oxidoreductase 1, to defend against oxidative damage and other stressful events (Aleksunes and Manautou, 2007). Nrf-2 activation in response to increased ROS can reduce ischemic brain injury (Shah et al., 2007; Zhang et al., 2014). Moreover, upregulation of Nrf-2 in neurons can protect against oxidative and excitotoxic insults (Son et al., 2010). These findings, along with our current results, suggest that the protective effects of PUR against oxidative stress, in part, occur via an increase in antioxidant expression. Moreover, an anti-apoptotic role of PUR was also present following HI injury. Since PUR or Shh agonists protect cortical neurons from apoptosis via a reduction of oxidative stress (Dai et al., 2011; Peterson and Turnbull, 2012), we speculated that the anti-apoptotic effects of PUR observed following HI injury, in part, may result from a reduction in oxidative stress through Nrf-2 activation. Additional work will be required to investigate the mechanisms through which PUR activates Nrf-2 in HI brain injury.

### PUR Attenuated Synaptic Damage Following HI Injury

During their initial developmental stages, animals subjected to HI injury show delayed neurite outgrowth and synaptogenesis, resulting in subsequent long-term memory impairments (Bona et al., 1997; Fathali et al., 2010). In rodents, cortical synaptic density is initially low in the newborn, but rapidly increases from PND10 until PND30, when equivalent levels to that observed in adults are present (Semple et al., 2013). Consistent with this previous study, we found that synaptic density increased from PND10 to PND21 and PND35 in each group. However, in mice subjected to HI, synaptic density at PND21 and PND35 reduced as compared with the Sham group. In addition, these reductions in synaptic densities were associated with decreased levels of synaptic proteins.

Synaptic proteins are directly involved in synaptic plasticity and related to cognitive function. A number of postsynaptic proteins including PSD95, PSD93, and synapse-associated protein 102 have been shown to be downregulated following early HI insult in neonatal mice (Shao et al., 2017). In addition, changes in synaptic morphology were associated with impaired recognition memory post-HI in neonatal rats (Ou-Yang et al., 2012). In the present study, reduced Syn and PSD95 expression levels were found in the cortex during early and advanced stages of development following HI insult. However, reduced Syn/PSD95 expression was not associated with behavioral abnormality in MWM test. Thus, the long-term memory impairments resulting from HI insult could not be attributed to the changes in Syn and PSD95 expression within the cortex.

Shh and its signaling receptor components are present in both presynaptic and postsynaptic terminals of the developing and adult CNS, suggesting that this pathway contributes to the synaptic plasticity of differentiated neurons (Petralia et al., 2011, 2012). It was reported that Shh agonist was able to improve learning and memory deficits in a Down syndrome mouse model (Das et al., 2013). Recent findings also found that stimulation of Shh release from neurons and astrocytes by bone marrow stromal cells could promote neurite outgrowth, synaptogenesis, and myelination (Ding et al., 2013). Our study showed that PUR treatment alleviated the synaptic loss and upregulated synaptic proteins expression following HI injury, which was associated with the involvement of the Shh pathway.

### PUR Restored Levels of Shh Signaling After HI

The endogenous Shh pathway participates in regenerative responses after damage to diverse brain regions, including the cerebral cortex and corpus callosum (Mierzwa et al., 2014). For example, a maximal activation of the Shh pathway was observed at 3 days after cortical freeze injury followed by a return to baseline levels at 14 days (Amankulor et al., 2009). Shh protein levels in the cortex were increased at 1–5 days after injury following a cortical stab wound (Sirko et al., 2013). In addition, the endogenous Shh pathway in the cortex and hippocampus was reported to be upregulated in response to stroke and ischemic insult (Sims et al., 2009; Chechneva et al., 2014). However, Shh expression in the cortex was downregulated in the early stages after experimental subarachnoid hemorrhage (Zuo et al., 2015; Hu et al., 2016). In the present study, the expression of Shh signaling including Shh and Ptch within brain was reduced following HI insult. PUR treatment produced an increased expression of Shh signaling 3 days post-HI, associated with reduced acute brain injury and improved long-term memory and learning deficits following HI. The significance of Shh signaling pathway was substantiated with the demonstration that Cyc treatment blocked these beneficial effects of PUR. Taken together, these data provided strong evidence indicating that PUR plays a protective role in HI-induced injury through activation of the Shh signaling pathway.

One limitation in this study was that ROS levels were measured using the DHE probes. In the presence of the superoxide anion, DHE is rapidly oxidized to oxyethidium, which binds DNA and emits light to monitor ROS activity (Huang et al., 2016). However, DHE is also easily oxidized by heme peroxidases. In the ischemic brain, heme peroxidase is elevated and released, which leads to an increase in ROS formation when monitored by ROS-sensitive fluorescent probes (Kim et al., 2016; Lu et al., 2018). To complement this, there are ongoing attempts to develop a new generation of ROS probes to measure ROS activity *in vivo*.

## Conclusion

In summary, PUR, a Shh pathway agonist, exerts protection against HI-induced injury in neonatal mice, as evidenced by its beneficial effects upon multiple parameters including acute brain injury, neuro-inflammtion, long-term memory deficits, and impaired synaptic function. Accordingly, PUR may serve as a promising new therapeutic drug candidate for HI injury, which can specifically and sensitively target the Shh signaling pathway to exert its beneficial effects.

## Data Availability Statement

The raw data supporting the conclusions of this article will be made available by the authors, without undue reservation, to any qualified researcher.

## Ethics Statement

The animal study was reviewed and approved by the Animal Ethical and Welfare Committee of Shandong University.

## Author Contributions

ZW made substantial contributions to study design, data interpretation, writing and revising of the manuscript, and final revision of the manuscript. DL made substantial contributions to rewriting and revising final manuscript, reading and editing proof, and performed data analysis and transmission electron microscopy. XB performed animal model, Western blot analysis, ROS content, and evaluation of neuropathological injury. WM performed Western blot analysis. DX, XC, HY, and JQ performed behavioral testing. HK, SY, and WC revised the manuscript. All authors read and approved the final draft of this manuscript.

## Conflict of Interest

The authors declare that the research was conducted in the absence of any commercial or financial relationships that could be construed as a potential conflict of interest.

## Supplementary Material

FIGURE S1 Schema for PUR treatment schedule, behavioral experiments, and tissue preparation. The pups on postnatal day 7 underwent right common carotid artery ligation, and hypoxia was induced (humidified 8% O_2_+92% N2 for 1.5 h). PUR or Cyc solutions were administered via intraperitoneal injection at 1, 2, and 3 days after HI insult. In the HI+PUR+Cyc group, Cyc was administered 30 min before PUR injection. The Sham and HI groups were treated with the same volume of the vehicle relative to body weight. Cyc, cyclopamine; HI, hypoxia-ischemia; ip, intraperitoneally; P, postnatal day; PUR, Purmorphamine; MWM, Morris water maze.

FIGURE S2 Effects of PUR on HI-induced neuronal damage. The representative transmission electron microscopy image of ipsilateral cortex. N = 4/group. Arrow indicates dissolved membrane. Asterisk points to swelling and vacuolization of mitochondria. Red arrowhead points to severe cytoplasmic edema. Scale bar = 2 μm. N, Nucleus; M, mitochondria; ER, rough endoplasmic reticulum.

FIGURE S3 Effects of PUR on HI-induced neuronal apoptosis. Three days following HI insult, sections form each group were subjected to TUNEL assay (green), counterstained with Dapi (blue). Scale bar = 50 μm. The percentage of TUNEL-positive cells was expressed as the number of positively stained apoptotic cells/the total cells counted. N = 4/group. Values represent the mean ± SD, ***p* < 0.01, ****p* < 0.001, according to ANOVA with Bonferroni correction.

FIGURE S4 The expression of Syn and PSD95 at 3, 14 and 28 days post-HI. The quantification of Syn and PSD95 within ipsilateral cortex was measured by Western blot at 3, 14, 28 days post HI. N = 3/group. Values represent the mean ± SD, **p* < 0.05, ***p* < 0.01, according to ANOVA with Bonferroni correction.

FIGURE S5 Effects of PUR administration on tissue loss at 14 days post-HI insult. Representative coronal sections of Nissl staining were obtained from different groups at 14 days after HI injury. Scale bar = 2000 μm. The quantification of tissue loss was determined with use of Image-Pro Plus 6⋅0. N = 6/group. Values represent the mean ± SD, **p* < 0.05, ***p* < 0.01, ****p* < 0.001, according to ANOVA with Bonferroni correction.

## Abbreviations

Cyc: cyclopamine
DAPI: 4′,6-diamidino-2-phenylindole dihydrochloride
HI: hypoxic-ischemic
Iba-1: ionized calcium binding adaptor molecule-1
MWM: Morris water maze
Nrf-2: nuclear factor-E2-related factor-2
OGD: oxygen-glucose deprivation
PND: postnatal day
PSD: postsynaptic density protein
Ptch: patched
PUR: purmorphamine
ROS: reactive oxygen species
Shh: sonic hedgehog
Smo: Smoothened
Syn: synaptophysin
TTC: triphenyltetrazolium chloride
