# Supplementary figures and images for: Purmorphamine Attenuates Neuro-Inflammation and Synaptic Impairments After Hypoxic-Ischemic Injury in Neonatal Mice via Shh Signaling
Source: Front Pharmacol. 2020 Mar 4;11:204. doi: 10.3389/fphar.2020.00204 (PMC7064623; doi:10.3389/fphar.2020.00204)

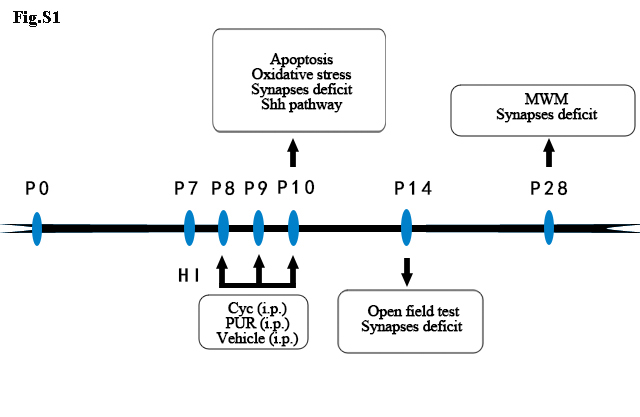

Supplement: FIGURE S1 — Schema for PUR treatment schedule, behavioral experiments, and tissue preparation. The pups on postnatal day 7 underwent right common carotid artery ligation, and hypoxia was induced (humidified 8% O2+92% N2 for 1.5 h). PUR or Cyc solutions were administered via intraperitoneal injection at 1, 2, and 3 days after HI insult. In the HI+PUR+Cyc group, Cyc was administered 30 min before PUR injection. The Sham and HI groups were treated with the same volume of the vehicle relative to body weight. Cyc, cyclopamine; HI, hypoxia-ischemia; ip, intraperitoneally; P, postnatal day; PUR, Purmorphamine; MWM, Morris water maze. [file Image_1.jpg]

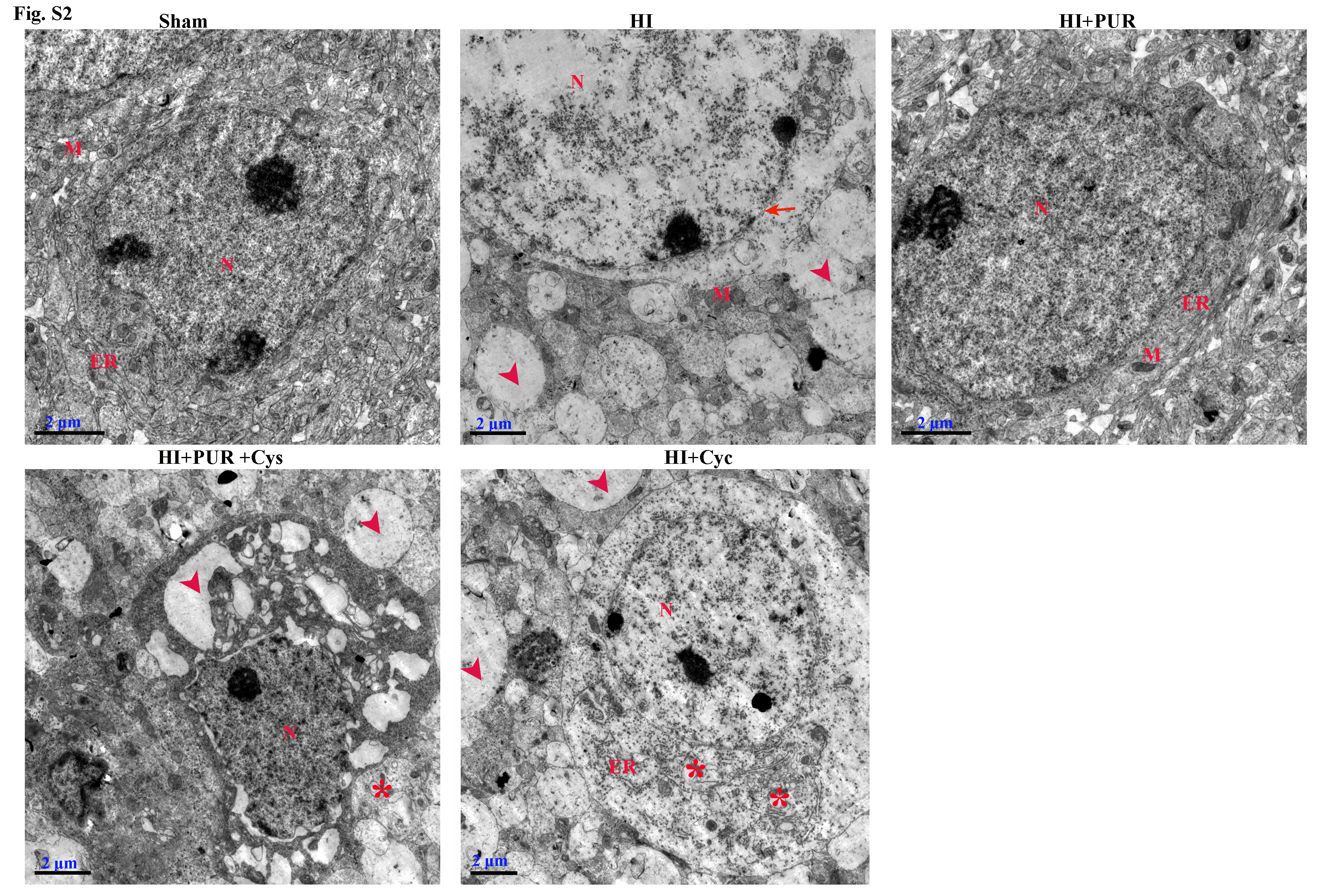

Supplement: FIGURE S2 — Effects of PUR on HI-induced neuronal damage. The representative transmission electron microscopy image of ipsilateral cortex. N = 4/group. Arrow indicates dissolved membrane. Asterisk points to swelling and vacuolization of mitochondria. Red arrowhead points to severe cytoplasmic edema. Scale bar = 2 μm. N, Nucleus; M, mitochondria; ER, rough endoplasmic reticulum. [file Image_2.jpg]

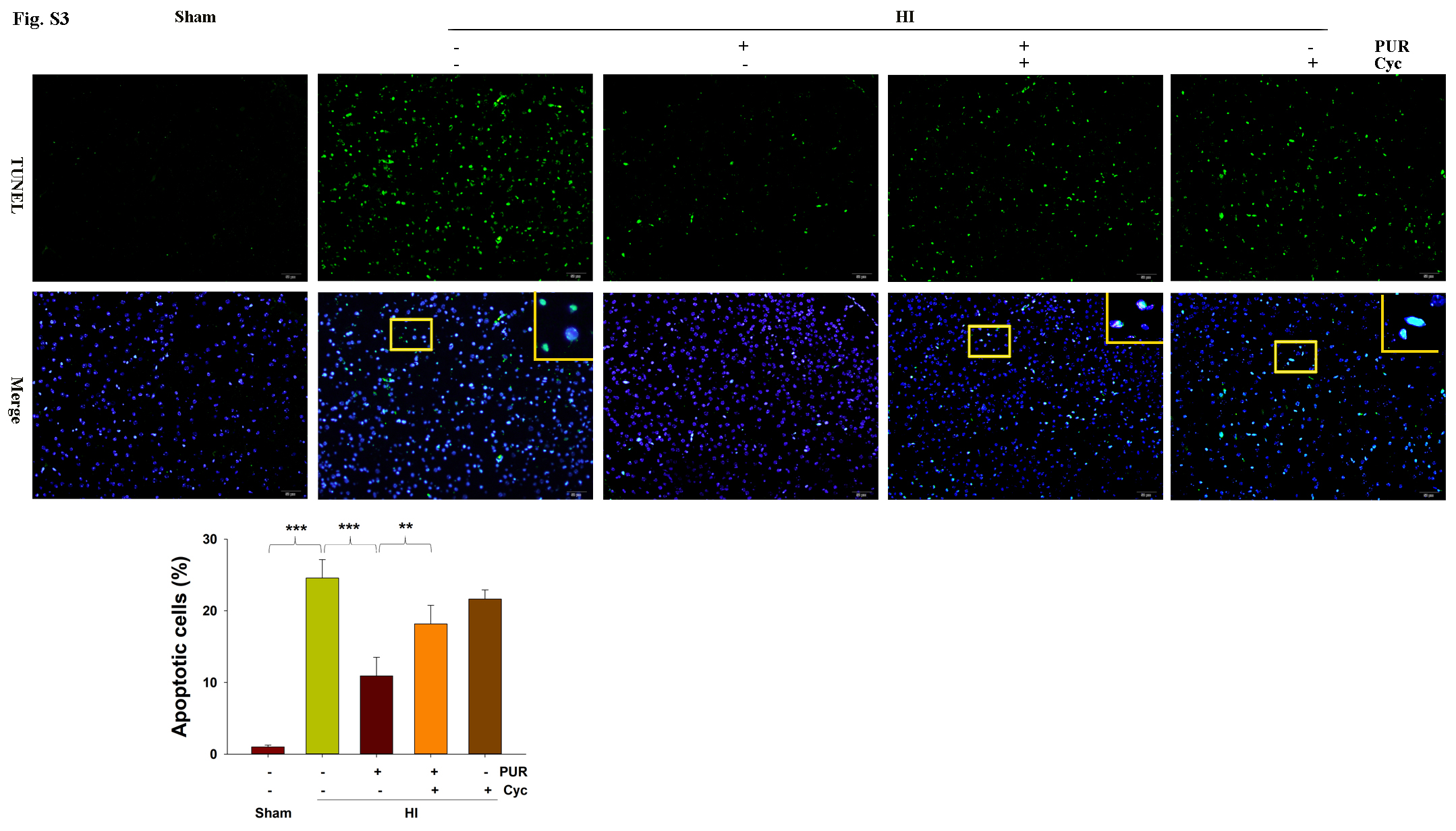

Supplement: FIGURE S3 — Effects of PUR on HI-induced neuronal apoptosis. Three days following HI insult, sections form each group were subjected to TUNEL assay (green), counterstained with Dapi (blue). Scale bar = 50 μm. The percentage of TUNEL-positive cells was expressed as the number of positively stained apoptotic cells/the total cells counted. N = 4/group. Values represent the mean ± SD, **p < 0.01, ***p < 0.001, according to ANOVA with Bonferroni correction. [file Image_3.jpg]

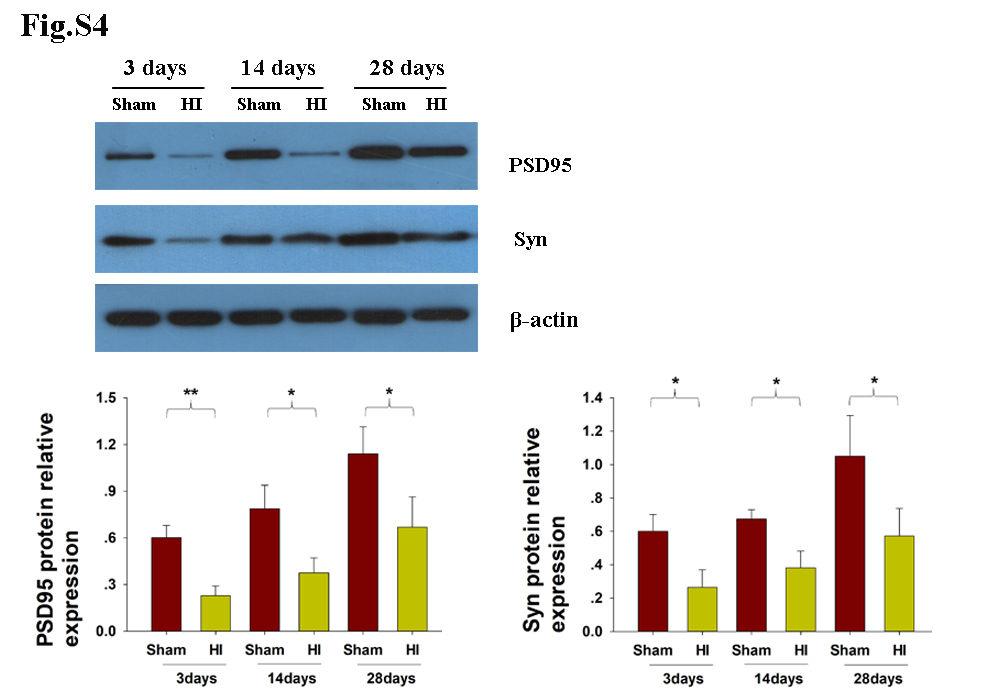

Supplement: FIGURE S4 — The expression of Syn and PSD95 at 3, 14 and 28 days post-HI. The quantification of Syn and PSD95 within ipsilateral cortex was measured by Western blot at 3, 14, 28 days post HI. N = 3/group. Values represent the mean ± SD, *p < 0.05, **p < 0.01, according to ANOVA with Bonferroni correction. [file Image_4.jpg]

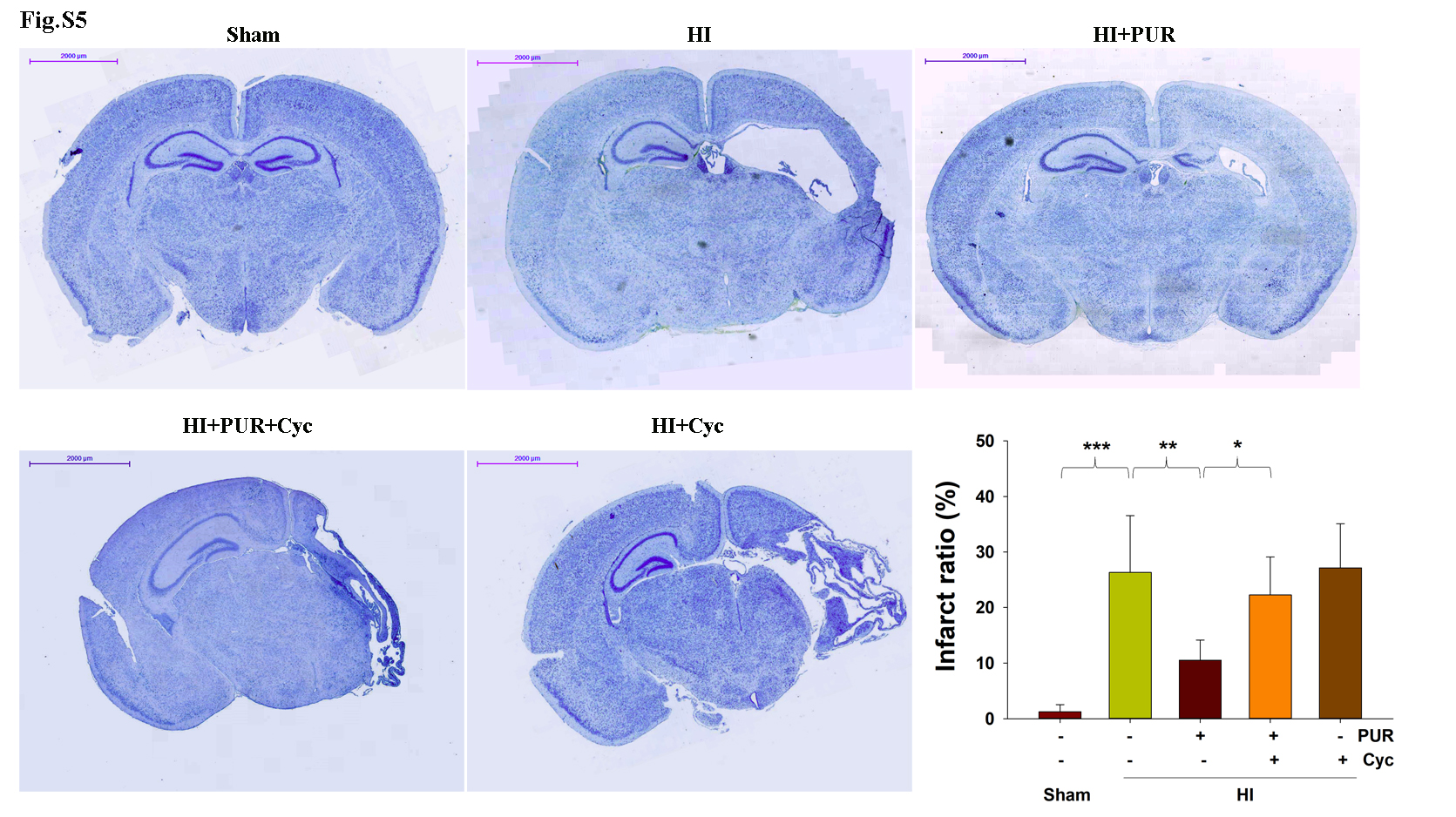

Supplement: FIGURE S5 — Effects of PUR administration on tissue loss at 14 days post-HI insult. Representative coronal sections of Nissl staining were obtained from different groups at 14 days after HI injury. Scale bar = 2000 μm. The quantification of tissue loss was determined with use of Image-Pro Plus 6⋅0. N = 6/group. Values represent the mean ± SD, *p < 0.05, **p < 0.01, ***p < 0.001, according to ANOVA with Bonferroni correction. [file Image_5.jpg]
